# Acid inhibitors and allergy: comorbidity, causation and confusion

**DOI:** 10.1038/s41467-020-17831-z

**Published:** 2020-08-07

**Authors:** Kewin Tien Ho Siah

**Affiliations:** 1 Division of Gastroenterology and Hepatology, Department of Medicine, National University Hospital, Level 10 Tower Block, 1E Kent Ridge Road, 119228 Singapore, Singapore; 2 Department of Medicine, Yong Loo Lin School of Medicine, National University of Singapore, 10 Medical Drive, 117597 Singapore, Singapore

**Keywords:** Gastroenterology, Health care

Arising from Jordakieva et al. *Nature Communications* 10.1038/s41467-019-10914-6 (2019)

The *Nature Communications* article by Jordakieva et al.^1^ has generated plenty of interest in both mainstream and social media. In short, the authors explored the prescription pattern of health insurance records in Austria from 2009 to 2013 and found an increased prescription of anti-allergic medications after the prescription of acid-inhibiting drug. Therefore, they inferred that gastric acid inhibition could lead to increased allergy risk.

Essentially, if a drug is prescribed before another drug, there is a chance that the first drug may influence the prescription of the second drug or potentially be responsible for the development of other diseases when using the second drug. In this case, the sequence of prescription is of utmost importance. However, we were not shown the prescription rate of acid inhibitors after anti-allergic medication. So, would the reverse also be true? There are many reasons why acid inhibitors and anti-allergic medication may be prescribed to the same patient. First, acid inhibitors and anti-allergic medications are two common classes of drugs that treat a multitude of diseases. Indication of acid inhibitors includes dyspepsia, peptic ulcer disease, gastroesophageal reflux disease, eosinophilic esophagitis, and Barrett’s esophagus. Antihistamines such as loratadine, cetirizine, and fexofenadine are medicines that are often used to relieve symptoms of allergies, such as hay fever, hives, allergic asthma, allergic rhinitis, atopic dermatitis, and insect bites.

Second, empirical therapy with acid inhibitors is common in the initial management of patients with functional dyspepsia (FD)^2^. Many epidemiological studies had shown an association between FD and allergic diseases, although the reason is still not known. Jones et al.^3^ examined 30,000 primary care medical records over a minimum of 5 years and reported an excess of atopic conditions in all functional gastro-intestinal disorder groups including FD. Koloski et al.^4^ reported an association between atopy and FD in a population-based study in Australia. Perhaps, proton pump inhibitors and allergy may be the missing link in the association between dyspepsia and allergic diseases. Therefore, if the association between acid inhibitors and anti-allergic medication is unidirectional, studies should then be investigating the prescription of anti-allergic medications after prokinetic agents.

Since medical school, medical professionals have always been cautioned against over-reliance on the findings of observational research, as they cannot establish cause-and-effect relationships. Schubert^5^ reported that many examples of suggested associations identified in observational studies were subsequently refuted in randomized controlled trials, which are known as the gold standard of clinical research. It is imperative to ask more questions and answer them scientifically through more trials before we create more anxiety and confusion.

## Data Availability

No datasets were generated during the current manuscript.
